# CagA Phosphorylation in Helicobacter pylori-Infected B Cells Is Mediated by the Nonreceptor Tyrosine Kinases of the Src and Abl Families

**DOI:** 10.1128/IAI.00349-16

**Published:** 2016-08-19

**Authors:** Linda M. Krisch, Gernot Posselt, Peter Hammerl, Silja Wessler

**Affiliations:** a Cancer Cluster Salzburg, Department of Molecular Biology, Division of Microbiology, Paris-Lodron University, Salzburg, Austria; b Department of Molecular Biology, Division of Allergy and Immunology, Paris-Lodron University, Salzburg, Austria; University of Illinois, Urbana

## Abstract

CagA is one of the most important virulence factors of the human pathogen Helicobacter pylori. CagA expression can be associated with the induction of severe gastric disorders such as gastritis, ulceration, gastric cancer, or mucosa-associated lymphoid tissue (MALT) lymphoma. After translocation through a type IV secretion system into epithelial cells, CagA is tyrosine phosphorylated by kinases of the Src and Abl families, leading to drastic cell elongation and motility. While the functional role of CagA in epithelial cells is well investigated, knowledge about CagA phosphorylation and its associated signal transduction pathways in B cells is only marginal. Here, we established the B cell line MEC1 derived from a B cell chronic lymphocytic leukemia (B-CLL) patient as a new infection model to study the signal transduction in B cells controlled by H. pylori. We observed that CagA was rapidly injected, strongly tyrosine phosphorylated, and cleaved into a 100-kDa N-terminal and a 40-kDa C-terminal fragment. To identify upstream signal transduction pathways of CagA phosphorylation in MEC1 cells, pharmacological inhibitors were employed to specifically target Src and Abl kinases. We observed that CagA phosphorylation was strongly inhibited upon treatment with an Src inhibitor and slightly diminished when the Abl kinase inhibitor imatinib mesylate (Gleevec) was applied. The addition of dasatinib to block c-Abl and Src kinases led to a complete loss of CagA phosphorylation. In conclusion, these results demonstrate an important role for Src and Abl tyrosine kinases in CagA phosphorylation in B cells, which represent druggable targets in H. pylori-mediated gastric MALT lymphoma.

## INTRODUCTION

Gastric mucosa-associated lymphoid tissue (MALT) lymphomas are low-grade, non-Hodgkin B cell lymphomas, which are closely associated with chronic inflammatory responses to infections with the class I carcinogen Helicobacter pylori (H. pylori) (1, 2). Successful H. pylori eradication therapy results in a complete regression in more than 70% of patients and is now the first-line strategy in treatment of MALT lymphomas (3, 4).

H. pylori expresses a large number of pathogenic factors that are implicated in the initiation and progression of gastric disorders (5). The cytotoxin-associated gene A (CagA) has attracted much attention as an oncogene, and its expression in H. pylori has been correlated with a number of H. pylori-dependent pathophysiological effects, including gastric cancer and MALT lymphoma (4, 6–8). In contrast to findings in H. pylori-positive gastritis patients, a majority of H. pylori-positive MALT lymphoma patients were serum positive for CagA (9, 10), leading to the hypothesis that CagA might be one causative factor in MALT lymphoma. Although the contribution of CagA to MALT lymphomas is still controversial (10, 11), a transgenic mouse model that systemically expressed CagA indicated a potential role for CagA in H. pylori-associated B cell lymphoma (8). However, the molecular mechanism of the involvement of CagA in MALT lymphoma formation remains elusive.

In gastric epithelial cells, the functions of H. pylori CagA have intensively been studied. In these cells, CagA is translocated into the cytoplasm via a type IV secretion system (T4SS). This process requires binding of the T4SS adhesin CagL to α_5_β_1_ integrins exposed on epithelial cells (12). Injected CagA is tyrosine phosphorylated in the C-terminally located Glu-Pro-Ile-Tyr-Ala (EPIYA) motifs EPIYA-A, EPIYA-B, and EPIYA-C/D by host cell kinases of the Src and Abl families (13–16). Src and Abl kinases function in a hierarchical and coordinated manner. Initially, c-Src phosphorylates the EPIYA-C or EPIYA-D motif (17). c-Src is then subsequently dephosphorylated and inactivated by a negative feedback loop triggered by the binding of phosphorylated CagA (p-CagA) to the C-terminal Src kinase (CSK) (18, 19). The tyrosine kinase c-Abl maintains EPIYA-A, EPIYA-B, and EPIYA-C/D phosphorylation of CagA at later time points at one or two sites (17). In the cytoplasm, translocated CagA can interact with several intracellular signaling proteins in a phosphorylation-dependent as well as phosphorylation-independent manner (20). As a consequence, CagA-mediated deregulation of downstream signaling pathways induces a drastic epithelial cell elongation (21–23).

Based on the knowledge that persistent bacterial colonization leads to the infiltration of neutrophils and lymphocytes into the mucosal epithelium (2, 24), it was hypothesized that H. pylori can directly interact with cells of the immune system. However, compared to information about gastric epithelial cells, the understanding of CagA functions in nonepithelial cells is rather limited. Previous studies were conducted in different types of professional phagocytes of the monocytic lineage, including THP-1, U937, J774A.1, and Josk-M. In these cell types, efficient T4SS-dependent CagA translocation and tyrosine phosphorylation have been demonstrated (25, 26). Further, a tyrosine-phosphorylated C-terminal CagA fragment was identified, indicating that CagA is rapidly cleaved into an N-terminal fragment exhibiting a molecular mass of approximately 100 kDa and a C-terminal part with a molecular mass of approximately 40 kDa with unknown functions (25, 26).

The high incidence of MALT lymphoma in persistent infections suggests that B cells might be directly infected by H. pylori as well. Recently, CagA translocation and tyrosine phosphorylation were observed in the B cell line BJAB (27). In B lymphocytes, CagA was shown to interact with the Src homology 2 domain tyrosine phosphatase (SHP-2) leading to the induction of mitogen-activated protein kinases and upregulation of the antiapoptotic proteins Bcl-2 (B cell lymphoma 2) and Bcl-X (27). Although these data indicate a possible contribution of CagA to the formation of MALT lymphoma, the signaling events leading to CagA tyrosine phosphorylation remained unclear.

In this study, we investigated CagA translocation and tyrosine phosphorylation in the B cell line MEC1, which is derived from a B cell chronic lymphocytic leukemia (B-CLL) patient (28). The nonreceptor tyrosine kinases Src and c-Abl functioned as potent CagA kinases in B cells, mediating phosphorylation of the EPIYA motifs in CagA. Tyrosine phosphorylation of CagA could efficiently be blocked by the Src and Abl inhibitor dasatinib, and thus Src and Abl represent possible targets in the treatment of CagA-positive MALT lymphoma.

## MATERIALS AND METHODS

### Cell culture and inhibitor treatment.

AGS, MEC1, and U937 cells were cultured in RPMI 1640 medium (Sigma, Germany) supplemented with 2 mM l-glutamine (Biowest, Germany) and 10% fetal calf serum (FCS) (Biowest, France) in a humidified 5% CO_2_ atmosphere at 37°C (Table 1). Adherent AGS cells were seeded in tissue culture dishes 48 h before infection and grown to 70% confluence. At 24 h prior to infection, medium was replaced by fresh serum-free medium. Suspension cells (MEC1 and U937) were harvested by centrifugation at 250 × *g* at 4°C for 5 min, and 5 × 10^6^ cells were seeded in 100-mm tissue culture dishes with serum-free medium at 24 h prior to infection. Where indicated, cells were pretreated with 10 μM PP2 to block Src kinases (Calbiochem, Austria), imatinib mesylate (STI-571; Gleevec) to block c-Abl, or dasatinib to block Src and Abl kinases (LC Laboratories, MA) for 30 min prior to infection experiments. Cells were routinely monitored using an inverted microscope (model CKX 41; Olympus).

**TABLE 1:** Mammalian cell lines

Cell line	Source (catalogue no.)^*a*^	Cell type	Growth property	Sample type and source (age)
AGS	ECACC (89090402)	Epithelial	Adherent	Gastric adenocarcinoma, Caucasian female (54 yr)
U937	ATCC (CRL-1593.2)	Monocyte	Suspension	Histiocytic lymphoma, Caucasian male (37 yr)
MEC1	DSMZ (ACC-497)	B cell	Suspension	Chronic B cell leukemia, Caucasian male (61 yr)

a ATCC, American Type Culture Collection (https://www.atcc.org); DSMZ, Deutsche Sammlung von Mikroorganismen und Zellkulturen GmbH (https://www.dsmz.de); ECACC, European Collection of Cell Cultures (https://www.phe-culturecollections.org.uk/collections/ecacc.aspx).

### Bacteria and infection experiments.

H. pylori wild-type (WT) strain (P12) (29), which expresses Western CagA harboring EPIYA-ABCC (14) and P12 Δ*cagA* have been described previously. H. pylori strains were cultured on agar plates containing 10% horse serum under microaerophilic conditions at 37°C for 48 h. For infection, bacteria were harvested in phosphate-buffered saline (PBS) and added to the host cells at a multiplicity of infection (MOI) of 100 for different time periods. As controls, equal amounts of PBS were added.

### MTT cell viability assay and statistical analysis.

Cell viability assays were performed with 1 × 10^4^ MEC1 cells in 96-well tissue culture plates in RPMI medium supplemented with 1% FCS. Cells were preincubated for 30 min with 10 μM PP2, 10 μM STI-571, 0.1 μM, or 10 μM dasatinib and then infected with H. pylori for 48 h at an MOI of 100 or were left uninfected. Cell viability was determined by incubating 10 μl of 5 mg/ml MTT [3-(4,5-dimethylthiazol-2-yl)2 2,5-diphenyl tetrazolium bromide; Sigma, Germany] at 37°C for 1 h. Cells were lysed in 110 μl of MTT lysis solution (0.1% NP-40, 0.04 N HCl in isopropanol), and the absorbance was measured at 570 nm in a plate reader (Tecan, Austria). Samples were prepared in quadruplicates. Cell survival of infected cells was normalized to the level of the respective noninfected controls treated with the same inhibitor. The mean of three independent experiments was used for statistical analysis. Significance of the observed effects between infected cells, untreated and treated with inhibitor, was calculated using Student's *t* test (paired, two-tailed).

### Plasmids and transient transfection.

A codon-optimized sequence of *cagA* from the H. pylori strain 26695 (P55980) was synthesized (GeneArt, Germany) and cloned into pCMV3-Tag expression vectors (Agilent Technologies, Austria) to create untagged CagA, C-terminally Flag-tagged CagA (CagA-Flag), and N-terminally Myc-tagged CagA (Myc-CagA). For the generation of phosphorylation-resistant (PR) mutants, the tyrosines Tyr^899^, Tyr^918^, and Tyr^972^ were replaced by alanines in the CagA-Flag (CagA-Flag-PR) fusion protein. For transient-transfection experiments, 1 × 10^6^ MEC1 cells were transfected with 5 μg of plasmid DNA using Lipofectamine LTX and Plus reagents according to the manufacturer's instructions (Life Technologies, Austria). Phosphorylation of ectopically expressed *cagA* was analyzed by cotransfecting 2.5 μg of pSGT-c-Abl wild type (WT), constitutively active pSGT-c-Abl PP (c-Abl P^242^E and P^249^E), or the kinase-dead (KD) variant pSGT-c-Abl KD (30). Since endogenous c-Abl levels in AGS cells are low (31), AGS cells were transfected with 5 μg of pSGT-c-Abl wild type in 100-mm tissue culture plates using polyethylenimine (PolyScience, USA).

### Immunoprecipitation, SDS-PAGE, and immunoblotting.

Adherent AGS cells were washed twice in PBS and harvested in lysis buffer (20 mM Tris-HCl, pH 7.5, 1 mM EDTA, 100 mM NaCl, 1% Triton X-100, 0.5% sodium deoxycholate, 0.1% SDS, 1× complete protease inhibitors [Roche, Germany], 1 mM Na_3_VO_4_, 1 mM sodium molybdate, 20 mM NaF, 10 mM sodium pyrophosphate, 20 mM β-glycerophosphate). Suspension cells (U937 and MEC1) were centrifuged at 250 × *g* at 4°C for 5 min after each washing step before lysis. Whole-cell lysates (WCLs) were cleared from debris by centrifugation, and the protein concentration was determined in a Bradford protein assay. Fifty micrograms of protein was separated by SDS-PAGE and transferred on nitrocellulose membranes. Membranes were blocked with Roti-Block (Carl Roth, Germany) and analyzed using the following antibodies: anti-phospho-tyrosine antibodies (PY99 [Santa Cruz, Germany] and 4G10 [Millipore, Germany]), anti-glyceraldehyde-3-phosphate dehydrogenase (GAPDH) antibody (Cell Signaling, Germany), anti-Myc-tag antibody (9B11; Cell Signaling), anti-Flag antibody (F1804; Sigma, Germany), anti-c-Abl antibodies (Ab3 [Calbiochem, Germany] and 24-11 [Santa Cruz, Germany]), anti-c-Src antibody (Santa Cruz, Germany), anti-phospho-c-Abl (p-Tyr^245^) antibody (Sigma, Germany), anti-phospho-SFK (p-Tyr^416^) antibody (Cell Signaling, Germany), and a phospho-specific CrkII antibody (p-Tyr^221^; Cell signaling, Germany). For the detection of the N-terminal region of CagA, a polyclonal CagA serum antibody was generated by immunization of rabbits with the recombinant N terminus of CagA (amino acids [aa] 1 to 900). A hybridoma cell line secreting a monoclonal CagA antibody to detect the phosphorylated and nonphosphorylated C terminus of CagA was produced by immunization of mice with the peptide SPEPIpYATIDDL conjugated to Escherichia coli beta-galactosidase as a carrier protein. To analyze c-Abl phosphorylation, c-Abl was immunoprecipitated by incubating 1 mg of whole-cell lysate with 5 μg of a monoclonal c-Abl antibody (Ab3; Calbiochem, Germany) overnight. Protein A and protein G Sepharose (GE Healthcare, Austria) were added for 2 h. Beads were washed three times in lysis buffer, and samples were boiled at 95°C for 7 min in sample buffer. Membranes were analyzed with a Molecular Imager ChemiDoc XRS+ system (Bio-Rad, Germany) or with an Odyssey Fc Imaging System (Li-Cor Biosciences, Austria) using anti-mouse IgG-horseradish peroxidase (HRP) and anti-rabbit IgG-HRP secondary antibodies (Sigma, Germany) and Amersham ECL Prime Western blotting reagent (GE Healthcare, Austria) or IRdye 800CW anti-mouse IgG and IRdye 800CW anti-rabbit IgG (Li-Cor Biosciences, Austria).

### *In vitro* kinase assay.

To analyze activity of upstream kinases, c-Abl or c-Src was immunoprecipitated from 500 μg of whole-cell lysate using 2 μg of anti-c-Src (Santa Cruz, Germany) or 2 μg of anti-c-Abl antibody (24-11; Santa Cruz, Germany). Beads were washed twice in lysis buffer and twice in kinase assay buffer (20 mM HEPES pH 7.4, 10 mM MgCl_2_, 10 mM MnCl_2_, and 2 mM dithiothreitol [DTT]). Immunoprecipitated c-Abl was analyzed separately by collecting one-fifth of the bead slurry directly after the washing steps. For *in vitro* kinase reactions of c-Src, H. pylori lysate containing CagA was used as a substrate. Briefly, H. pylori was sonicated in kinase buffer and centrifuged at 20,000 × *g*. Ten micrograms of cleared H. pylori WT or H. pylori Δ*cagA* lysate was added as a kinase substrate. For *in vitro* kinase reactions of c-Abl, 1 μg of recombinant glutathione *S*-transferase (GST)-CrkII WT (aa 120 to 225) (32) was added to the washed antigen-coupled beads together with 250 μM ATP and incubated for 30 min at 30°C and 1,000 rpm on a thermomixer (Eppendorf, Germany). Recombinant GST-CrkII (aa 120 to 212) was included as a negative control (32). To stop the reaction, 4× sample buffer was directly added to the samples and immediately boiled at 95°C for 7 min and subjected to Western blot analysis.

## RESULTS

### Translocation and tyrosine phosphorylation of H. pylori CagA in B cells.

**FIG 1:**
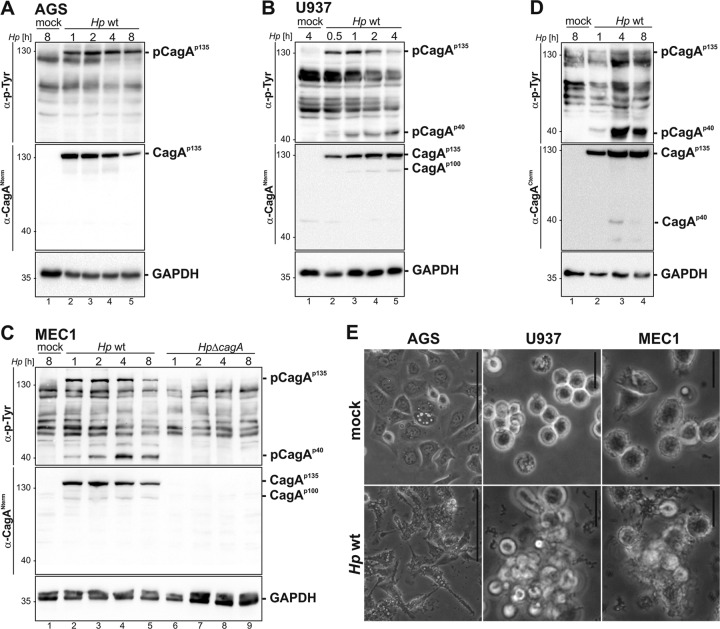
Injection and phosphorylation of CagA in H. pylori-infected AGS, U937, and MEC1 cells. AGS cells (A) or U937 cells (B) were colonized with an H. pylori (*Hp*) wild-type strain at an MOI of 100 for the indicated time periods or remained uninfected (mock). (C) MEC1 cells were colonized with the WT strain or an isogenic *cagA* mutant (Δ*cagA* strain) at an MOI of 100 for the indicated time periods or remained uninfected (mock). Whole-cell lysates were analyzed for phosphorylated CagA (p-CagA) using an anti-phospho-tyrosine antibody (α-p-Tyr). Total CagA (CagA^p135^, CagA^p100^, and CagA^p40^) was detected using anti-CagA antibody recognizing the N-terminal part of CagA (α-CagA^Nterm^) or an antibody directed against the C terminus of CagA (α-CagA^Cterm^). GAPDH is shown as a loading control. (D) MEC1 cells were infected with the WT strain at an MOI of 100 for the indicated time periods or left uninfected (mock). Cells were boiled directly in sample buffer. (E) AGS, U937, and MEC1 cells were infected with the WT strain or left untreated (mock). Phase-contrast microscopy was performed after 4 h of infection. Bar, 50 μm.

CagA translocation and phosphorylation are well characterized in gastric epithelial AGS cells (33, 34) and have also been detected in several myeloid cell lines and phagocytic cell types (25, 26). To investigate the H. pylori-dependent signal transduction pathways that possibly contribute to the induction and progression of MALT lymphoma, MEC1 cells derived from B cell chronic lymphocytic leukemia (B-CLL) were established as a new infection model for H. pylori. As the CagA phosphorylation patterns differ in various cell types, CagA injection and tyrosine phosphorylation were compared in H. pylori-infected gastric epithelial cells (AGS), monocytic cells (U937), and B cells (MEC1). AGS cells were infected with H. pylori wild type (WT) for the time periods indicated in Fig. 1A, and translocation of CagA was detected by an anti-phospho-tyrosine antibody. H. pylori rapidly translocated CagA into AGS cells within 1 h of infection, as monitored by the detection of tyrosine-phosphorylated CagA (Fig. 1A, top panel). Correspondingly, full-length CagA exhibiting a molecular mass of approximately 135 kDa (CagA^p135^) was observed (Fig. 1A, middle panel). Detection of the housekeeping protein glyceraldehyde-3-phosphate dehydrogenase (GAPDH) indicated equal protein loading (Fig. 1A, bottom panel). Studies describing CagA injection into monocytic cells, such as THP-1 or U937 cells, showed a different pattern of CagA translocation (25, 26). In line with these reports, translocated p-CagA^p135^ appeared within 30 min postinfection and declined after 8 h of infection with H. pylori (Fig. 1B, top panel). In contrast to results in epithelial cells, CagA^p135^ was fragmented into a 100-kDa N-terminal CagA^p100^ (Fig. 1B, middle panel) and a prominent tyrosine-phosphorylated 40-kDa C-terminal CagA^p40^ (p-CagA^p40^) (Fig. 1B, top panel). Translocation of CagA into human B lymphocytes has previously been shown in the human B lymphoma cell line BJAB (27). However, the signal transduction pathways leading to CagA phosphorylation and fragmentation of (p)CagA have not been analyzed yet. Therefore, MEC1 cells were infected with H. pylori wild type (WT) as indicated in Fig. 1C, and translocation and phosphorylation of CagA were examined. In fact, p-CagA^p135^ was detected after 1 h, demonstrating efficient CagA translocation and phosphorylation in the B cell line MEC1, which is perfectly in line with a previous study showing CagA injection in the human B lymphoma cell line BJAB (27). In addition to these recent findings, we observed that H. pylori-injected CagA was also fragmented in a CagA^p100^ and a CagA^p40^ part. The CagA^p40^ harbored the tyrosine-phosphorylated EPIYA motifs (Fig. 1C, top and middle panel), as described for cells of monocytic origin like U937 or THP-1 (25, 26). Additionally, MEC1 cells were infected with an isogenic *cagA* deletion mutant (Δ*cagA* strain) which resulted in a complete loss of (p)CagA^p135^, CagA^p100^, and p-CagA^p40^ signals (Fig. 1C, top and middle panels). To exclude the possibility that CagA phosphorylation and fragmentation occur in the lysates after cell disruption, we directly lysed H. pylori-infected cells in reducing SDS sample buffer. Still, equal amounts of CagA^p135^ and p-CagA^p40^ could be detected (Fig. 1D), demonstrating that CagA phosphorylation and fragmentation require T4SS-mediated translocation. Changes in cell morphology of H. pylori-infected AGS, U937, and MEC1 cells were monitored by phase-contrast microscopy (Fig. 1E). AGS cells strongly elongated in response to H. pylori infections, as expected. H. pylori-colonized U937 cells formed multicell aggregates, which have previously been attributed to the T4SS-dependent CagA translocation process (35). Uninfected MEC1 cells grew in suspension and showed a typical round morphology. At 4 h of postinfection, MEC1 cells formed aggregates comparable to those of U937 cells (Fig. 1D). In conclusion, these data show that H. pylori CagA was efficiently injected into MEC1 cells, followed by its cleavage into two fragments. This demonstrates that MEC1 cells represent a suitable infection model to study CagA signal transduction pathways in B cells.

### Overexpression of CagA in MEC1 cells.

**FIG 2:**
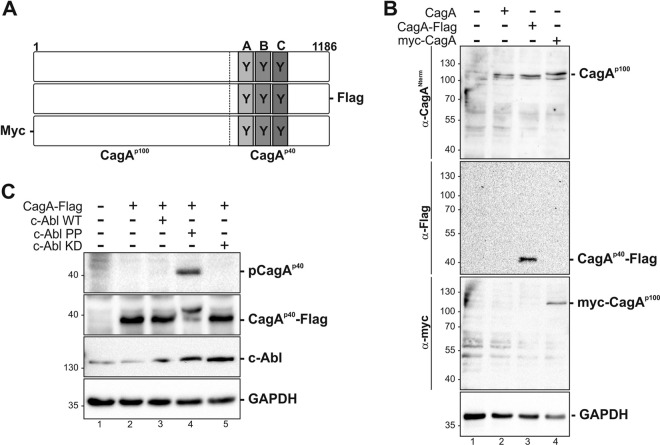
Detection of ectopically expressed CagA in MEC1 cells. (A) Schematic overview of the untagged, Flag-tagged, and Myc-tagged *cagA* constructs with the EPIYA motifs A, B, and C (gray boxes). (B) MEC1 cells were transfected with 5 μg of the indicated *cagA* WT constructs (+) or remained untransfected (−). Protein lysates were analyzed with an anti-CagA antibody to detect the N-terminal region of CagA (CagA^p100^), anti-Flag antibody to detect the C-terminally Flag-tagged CagA (CagA^p40^-Flag), and anti-Myc-tag antibody to detect the N-terminally Myc-tagged CagA (Myc-CagA^p100^). Anti-GAPDH was applied as a loading control. (C) MEC1 cells were transfected with CagA-Flag WT alone or cotransfected with cDNAs encoding c-Abl wild type (c-Abl WT), constitutively active c-Abl (c-Abl PP), or a kinase-dead variant of c-Abl (c-Abl KD), as indicated (+), or remained untransfected (−). The phosphorylated C-terminal CagA fragment (p-CagA^p40^) was analyzed with anti-phospho-tyrosine antibody. An anti-Flag antibody was used to detect the C-terminally Flag-tagged CagA (CagA^p40^-Flag). c-Abl and GAPDH were applied as expression and loading controls.

A putative cleavage site in the CagA molecule has been suggested in the C-terminal region of CagA, leading to the formation of a 100-kDa N-terminal and a 40-kDa C-terminal fragment, which harbors the EPIYA motifs (36) that are tyrosine phosphorylated by kinases of the Src and Abl families in AGS cells (14, 15, 17). To analyze the origin of the fragments in MEC1 cells, we transfected expression vectors harboring a codon-optimized CagA sequence. We overexpressed CagA, C-terminally Flag-tagged CagA (CagA-Flag), and N-terminally Myc-tagged CagA (Myc-CagA) (Fig. 2A). As indicated, CagA expression and fragmentation in transfected MEC1 cells were analyzed using antibodies recognizing either the N-terminal region of CagA (Fig. 2B, first panel), the C-terminally located Flag tag (Fig. 2B, second panel), or the N-terminally located Myc tag (Fig. 2B, third panel). Using the tag-specific antibodies, we could confirm the C-terminal origin of CagA^p40^ and the N-terminal derivation of CagA^p100^ (Fig. 2B). Interestingly, we could not detect the full-length CagA protein in transfected MEC1 cells, which suggests rapid and efficient CagA fragmentation in MEC1 cells. However, the fragmentation patterns of both CagA translocated by H. pylori and ectopically expressed CagA in MEC1 cells appear identical. We could not detect tyrosine phosphorylation of ectopically expressed CagA (p-CagA^p40^) in unstimulated cell lysates (Fig. 2C, first panel, lane 2). Hence, c-Abl wild type (c-Abl WT), a constitutively active c-Abl construct (c-Abl PP), or a kinase-dead variant of c-Abl (c-Abl KD) (30) was cotransfected with C-terminally Flag-tagged CagA (CagA-Flag). Phosphorylated CagA^p40^ could be detected only after cotransfecting CagA-Flag and c-Abl PP (Fig. 2C, first panel, lane 4), which is in contrast to findings of previous studies showing that ectopic CagA is constitutively phosphorylated in AGS cells (37, 38). Using a Flag tag-specific antibody (Fig. 2C, second panel), an obvious shift of the molecular mass of CagA^p40^-Flag was detected, indicating efficient tyrosine phosphorylation by c-Abl PP. Overexpression of c-Abl was verified using an antibody against c-Abl (Fig. 2C, third panel). These data indicate that phosphorylation of ectopic CagA requires activated nonreceptor tyrosine kinases and that it is efficiently cleaved in MEC1 cells.

### Activity of host cell kinases during H. pylori infection in MEC1 cells.

**FIG 3:**
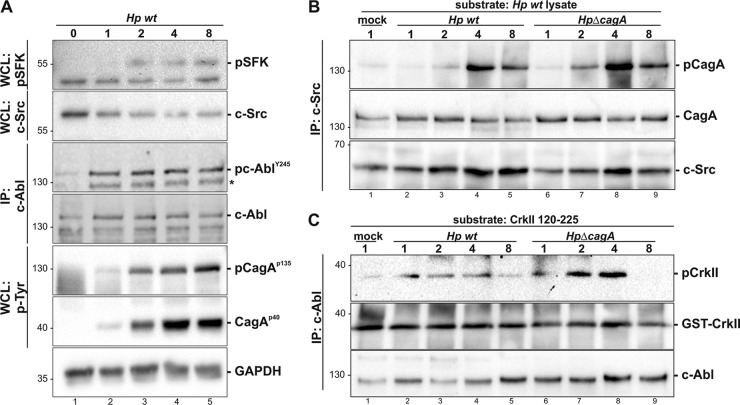
Activation of Src family kinases and c-Abl in H. pylori-infected MEC1 cells. (A) MEC1 cells were infected with an H. pylori (*Hp*) wild-type strain at an MOI of 100 for the indicated time periods. Whole-cell lysates (WCLs) were analyzed for phosphorylated Src family kinases (p-SFK), c-Src, and phosphorylated CagA (p-CagA^p135^ and p-CagA^p35^). To detect phosphorylated c-Abl, c-Abl was immunoprecipitated (IP) from 1 mg of protein lysate using a monoclonal c-Abl antibody. Phosphorylated c-Abl (p-c-Abl^Y245^) and c-Abl were analyzed by immunoblotting. GAPDH is shown as a loading control. Asterisks indicate a fragment of c-Abl. (B) MEC1 cells were infected with an H. pylori wild-type strain at an MOI of 100 for the indicated time periods. c-Src was immunoprecipitated from 500 μg of protein lysate using a polyclonal c-Src antibody. An *in vitro* kinase assay was performed with 10 μg of WT lysate containing CagA protein as a substrate. Phosphorylated CagA (p-CagA), CagA, and c-Src were analyzed by immunoblotting. (C) MEC1 cells were infected with an H. pylori wild-type strain at an MOI of 100 for the indicated time periods. c-Abl was immunoprecipitated from 500 μg of protein lysate using a monoclonal c-Abl antibody. *In vitro* kinase assays were performed using 1 μg of recombinant GST-tagged CrkII (aa 120 to 225) as a substrate. Phosphorylated CrkII (p-CrkII) and GST-CrkII were detected by immunoblotting. Aliquots of immunoprecipitated c-Abl, prior to the *in vitro* phosphorylation assay, were analyzed for equal c-Abl amounts.

In gastric epithelial cells, Src family kinases (SFKs) and c-Abl phosphorylate CagA in a hierarchical manner (14, 15, 17). To investigate the upstream kinases leading to CagA phosphorylation in B cells, we analyzed SFK and c-Abl phosphorylation and activity. Hence, we infected MEC1 cells with an H. pylori wild-type (WT) strain for the time periods indicated in Fig. 3A. Since c-Src activity is regulated by phosphorylation at Tyr^416^ in the activation loop of the kinase domain or equivalent sites in other SFK members, we monitored SFK activation by the detection of p-SFK^Y416^. The phosphorylation level of a 55-kDa SFK member increased in whole-cell lysates (WCLs). Additionally, the 60-kDa SFK member c-Src was detected and decreased during infection with H. pylori (Fig. 3A, first and second panels). Although we did not determine the phosphorylation status of different SFKs in MEC1 cells, we conclude from these data that individual SFK members are immediately regulated in H. pylori infections. In gastric epithelial cells, Src kinases are dephosphorylated after 2 h of infection, and CagA phosphorylation is then maintained by Abl kinases (14, 15, 17). To investigate whether SFKs and c-Abl also share a coordinated role in MEC1 cells, c-Abl activity was analyzed by immunoprobing of c-Abl Tyr^245^ phosphorylation (pc-Abl^Y245^) (14, 39). pc-Abl^Y245^ increased in H. pylori-infected cells, indicating that c-Abl is activated after 1 h to 8 h postinfection (Fig. 3A, third panel). The cellular amount of c-Abl increased slightly (Fig. 3A, fourth panel), as described previously for gastric epithelial cells and MALT lymphoma (14, 39). As controls, p-CagA^p135^, p-CagA^p40^, and GAPDH were detected (Fig. 3A, fifth to seventh panels). To investigate the kinase activity of c-Src in more detail, c-Src was immunoprecipitated from WCLs of MEC1 cells, which were infected with either H. pylori WT or H. pylori Δ*cagA*, as indicated on Fig. 3B. Efficient CagA translocation and cleavage have been detected in WCLs (see Fig. S1A in the supplemental material). As a kinase substrate for c-Src, CagA was incubated with immunoprecipitated c-Src (Fig. 3B, lanes 1 to 9). Src-mediated phosphorylation of CagA was detected using an anti-phospho-tyrosine antibody (Fig. 3B, top panel). Corresponding to SFK phosphorylation (Fig. 3A), the kinase activity of c-Src increased after 2 to 4 h of infection with H. pylori wild type (Fig. 3B, lanes 2 to 5). Infection with an H. pylori Δ*cagA* deletion mutant induced a stronger activity of c-Src (Fig. 3B, lanes 6 to 9). Equal CagA substrate amounts (Fig. 3B, middle panel) and immunoprecipitated c-Src (Fig. 3B, bottom panel) were demonstrated as controls. Finally, the efficiency of c-Src immunoprecipitation (see Fig. S1B, lanes 1, 2, and 5, in the supplemental material) and of CagA phosphorylation have been shown (see Fig. S1B, lanes 1, 4, and 5). Additionally, we analyzed c-Abl kinase activity in MEC1 cells, which were infected with H. pylori wild-type or a *cagA* deletion mutant for the indicated time periods (Fig. 3C; see also Fig. S2A in the supplemental material). c-Abl was immunoprecipitated prior to the *in vitro* phosphorylation assay using recombinant GST-CrkII as a substrate (32) (Fig. 3C). Phosphorylated GST-CrkII (p-CrkII) was detected using a phospho-specific CrkII antibody. Interestingly, c-Abl was activated already at 1 h postinfection, and its activity was reduced at later time points (Fig. 3C, lanes 1 to 5). Compared to infection with H. pylori wild type, infection with a *cagA* deletion mutant of H. pylori induced strong c-Abl activity (Fig. 3C, lanes 6 to 9). In parallel, the specificity of CrkII phosphorylation by immunoprecipitated c-Abl was validated using a truncated GST-CrkII substrate that lacked tyrosine 221 (see Fig. S2B, lane 1, in the supplemental material), and the specificity of c-Abl immunoprecipitation was controlled by using a preimmune serum (PIS) instead of an anti-c-Abl antibody (see Fig. S2C). The substrates GST-CrkII wild type, the truncated GST-CrkII protein (see Fig. S2B, lanes 3 to 4), and lysates from noninfected MEC1 cells (see Fig. S2B, lane 5) were examined as further controls. In summary, these data imply that the activities of c-Src and c-Abl were activated in H. pylori-infected MEC1 cells but are negatively regulated by CagA translocation into MEC1 cells.

### Phosphorylation of CagA in MEC1 cells is mediated by SFK and c-Abl kinases.

**FIG 4:**
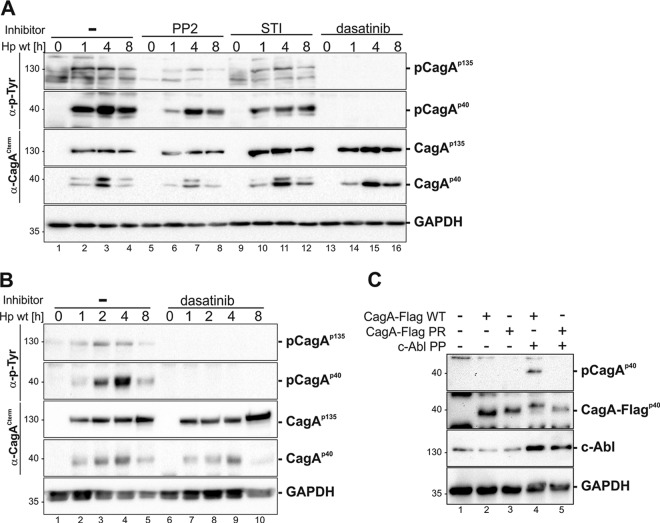
Tyrosine phosphorylation of H. pylori CagA by coordinated Src and c-Abl activities. (A) MEC1 cells were treated with 10 μM PP2, 10 μM STI-571, and 10 μM dasatinib prior to infection or remained untreated (−). (B) U937 cells were pretreated with 10 μM dasatinib or remained untreated (−). Cells were infected with an H. pylori (*Hp*) wild-type strain for the indicated time periods. Whole-cell lysates were analyzed by immunoblotting using an anti-phospho-tyrosine (α-p-Tyr) antibody to detect phosphorylated full-length CagA (p-CagA^p135^) and a C-terminal CagA fragment (p-CagA^p40^). A monoclonal anti-CagA antibody recognizing the C-terminal part of CagA (α-CagA^Cterm^) was applied to verify full-length and fragmented CagA (CagA^p135^ and CagA^p40^). As a loading control, the blot was reprobed with an anti-GAPDH antibody. (C) MEC1 cells were cotransfected with cDNAs encoding CagA-Flag wild-type (WT) or phospho-resistant CagA (CagA-Flag PR) with a plasmid expressing constitutively active c-Abl (c-Abl PP), as indicated. Protein lysates were analyzed for the phosphorylated C-terminal CagA fragment (p-CagA^p40^). To detect the C-terminally Flag-tagged CagA (CagA^p40^-Flag), an anti-Flag antibody was used. c-Abl and GAPDH were applied as controls.

After demonstrating efficient CagA injection and activation of SFK and c-Abl in MEC1 cells, we continued to determine whether SFK and Abl kinases represent the responsible upstream kinases for CagA phosphorylation. As specific pharmacological inhibitors, the following were preincubated with MEC1 cells prior to infection with H. pylori: PP2 blocking SFKs (40); STI-571 inactivating Abl, platelet-derived growth factor receptor (PDGFR), and c-Kit kinases (41); and dasatinib targeting SFK, Abl, c-Kit, PDGFR-α, PDGFR-β, and ephrin receptor kinase (42). Compared to levels in untreated MEC1 cells, p-CagA^p135^ was strongly inhibited in cells pretreated with PP2 and slightly inhibited when STI-571 was applied. Addition of dasatinib led to a complete loss of p-CagA^p135^ (Fig. 4A, first panel). Similarly, phosphorylation of CagA^p40^ was reduced after the addition of PP2 or STI-571 but was completely abolished upon treatment with dasatinib (Fig. 4A, third panel). To test the hypothesis of whether CagA fragmentation depends on CagA phosphorylation, we analyzed the formation of CagA^p40^ in dasatinib-treated MEC1 cells using a monoclonal anti-CagA antibody that recognizes the C-terminal part of CagA irrespective of its phosphorylation (Fig. 4A, fourth panel). The results corroborate the inhibition of CagA^p40^ phosphorylation by dasatinib since the shift in the molecular mass of CagA^p40^ due to its phosphorylation was prevented (Fig. 4A, fourth panel). This indicates (i) that CagA fragmentation is independent of tyrosine phosphorylation and (ii) that CagA phosphorylation requires the coordinated activity of both SFKs and c-Abl. Experiments were also performed using lower concentrations of dasatinib, showing its high efficiency in preventing CagA phosphorylation (see Fig. S3 in the supplemental material). In addition, we demonstrated that CagA phosphorylation in H. pylori-infected U937 cells was also strictly dependent on SFK and c-Abl activities. Comparable to the results obtained in MEC1 cells, CagA phosphorylation was completely inhibited after pretreatment with dasatinib (Fig. 4B). To finally confirm that processing of CagA is independent of tyrosine phosphorylation, we cotransfected C-terminally Flag-tagged wild-type CagA (CagA-Flag WT) or the corresponding phosphorylation-resistant mutant (CagA-Flag PR) with constitutively active c-Abl (c-Abl PP) (Fig. 4C). As expected, tyrosine-phosphorylated p-CagA^p40^ could be detected only in cells transfected with CagA-Flag WT and was absent in cells transfected with the phospho-resistant CagA protein. Using an anti-Flag-tag antibody (Fig. 4C, second panel), we verified that CagA processing is not associated with its tyrosine phosphorylation.

### Inhibition of CagA kinases in H. pylori-infected MEC1 cells results in reduced cell death.

**FIG 5:**
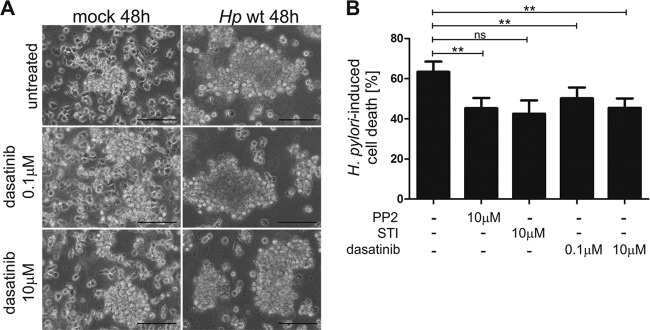
SFK and c-Abl activities play a crucial role in cell death of H. pylori-infected MEC1 cells. (A) MEC1 cells were treated with 0.1 μM or 10 μM dasatinib prior to infection or left untreated. Phase-contrast microscopy was performed after 48 h of infection. Bar, 100 μm. (B) MEC1 cells were pretreated with 10 μM PP2, 10 μM STI-571, or 0.1 μM or 10 μM dasatinib or remained untreated (−). After infection for 48 h, cell proliferation was measured by an MTT assay. H. pylori-infected cell levels were normalized to those of the respective noninfected controls treated with the same inhibitor. Results represent the means ± standard deviations of three independent experiments performed in quadruplicates. **, *P* < 0.01; ns, not significant.

To investigate the functional consequences of SFK/c-Abl inhibition, we further analyzed the cell aggregation upon H. pylori infection. H. pylori induced the formation of homotypic aggregates and multicell complexes, which were not affected by the pretreatment with 100 nM or 10 μM dasatinib (Fig. 5A). These data also suggest that MEC1 cell aggregation is independent of CagA phosphorylation, which could be verified in experiments using an isogenic *cagA* deletion mutant (data not shown). As Src and Abl kinases are closely associated with cell survival (43, 44), the effect of CagA kinases on H. pylori-induced cell death was investigated. H. pylori-infected MEC1 cells were pretreated with the inhibitors PP2 (SFK), STI-571 (c-Abl), or dasatinib (SFK and c-Abl) and analyzed by an MTT assay. To monitor the effects of the inhibitors on H. pylori-mediated cell death, mean values of H. pylori-infected cells were normalized to those of noninfected cells. Approximately 60% cell death was induced in reaction to infection with H. pylori (Fig. 5B), which was decreased by 18% (*P* = 0.0038) after SFK inhibition by PP2. Inhibition of c-Abl by STI-571 resulted in a 21% reduction (*P* = 0.1400) of cell death in H. pylori-infected MEC1 cells. Similar results were obtained using 0.1 μM (*P* = 0.0081) and 10 μM dasatinib (*P* = 0.0051) (Fig. 5B). In conclusion, SFK and c-Abl kinase activities contribute to H. pylori-mediated cell death. These data support the hypothesis that CagA phosphorylation interferes with survival pathways and might contribute to the transforming capacity of H. pylori infections.

## DISCUSSION

The expression of the pathogenic factor CagA can be correlated with a number of H. pylori-associated disorders, such as gastric cancer or MALT lymphoma. CagA translocates through the T4SS pilus into the cytoplasm of infected cells, where it localizes to the plasma membrane, is tyrosine phosphorylated through host kinases, and induces elongation of epithelial cells (22, 34). In this context, SFKs and c-Abl were identified in gastric epithelial cells to phosphorylate CagA at the EPIYA motifs in the C terminus, which directly promote epithelial cell elongation (14, 15, 17). Since colonization of the epithelium with H. pylori leads to an infiltration of lymphocytes in the gastric mucosa (45), it was hypothesized that lymphocytes can be directly targeted by H. pylori *in vivo*. In this study, we employed the cell line MEC1 derived from a B-CLL patient (28) as a model to study the influence of H. pylori on B cell functions. In accordance with a recent report on the human B lymphoma cell line BJAB (27), H. pylori translocated CagA into MEC1 cells, as monitored by the detection of the phosphorylated CagA^p135^. In the present study, we additionally demonstrate CagA fragmentation in B cells and detected tyrosine-phosphorylated p-CagA^p135^, p-CagA^p40^, and nonphosphorylated CagA^p100^. To date, CagA fragmentation has been described exclusively in phagocytic cells, including U937, THP-1, J774A.1, and Josk-M cells (25, 26), but it is still unknown whether CagA fragmentation has a significant biological role in these cells. Phenotypically, U937 cells aggregated after infection with H. pylori in a T4SS-dependent manner by the upregulation and recruitment of ICAM-1 (intercellular adhesion molecule 1) to the surface of U937 cells (35), indicating that a functional T4SS and possibly CagA are implicated in this process. In nonepithelial cells, CagA-dependent signal transduction pathways are not well investigated. In BJAB cells, CagA interacts with the SHP-2 phosphatase and activates extracellular signal-regulated kinase 1 and 2 (ERK1/2) and p38 kinases, which are implicated in proliferative cell responses. Since the antiapoptotic proteins Bcl-2 and Bcl-X are upregulated in BJAB cells, the authors concluded that translocated CagA promotes B cell survival (27). This effect was partly reproduced in B1 lymphocytes transduced by retroviral vectors carrying the *cagA* gene (46). However, ectopic overexpression of the EPIYA motif harboring part of CagA in the interleukin-3 (IL-3)-dependent mouse pro-B cell line BaF/6-1 leads to an inhibition of proliferation, which is caused by a significant delay in G_1_-S transition. The decrease of proliferation requires inhibition of the IL-3/Jak2/Stat5 (Janus kinase/signal transducer and activator of transcription) and p53 signaling pathways, and is independent of CagA tyrosine phosphorylation (47). Although we did not investigate the Jak/Stat signaling pathway, our data imply that tyrosine kinase signaling by SFK and c-Abl is implicated in the decrease of B cell proliferation. In fact, the impact of the nonreceptor tyrosine kinases in cell proliferation, cell cycle, and apoptosis is well established (43, 44). Therefore, we conclude that H. pylori activates SFK- and c-Abl-dependent signal transduction pathways to control B cell proliferation. In this context, it is still speculative if CagA fragments are involved in this process.

Injection and tyrosine phosphorylation have been repeatedly demonstrated in nonepithelial cells, while the identity of CagA kinases was not investigated. We showed that host cell kinases of the Src and Abl families play a crucial role in the phosphorylation of translocated CagA, as previously described for gastric epithelial cells (13–15, 17). However, the kinetics of kinase activity in MEC1 cells differed from that in gastric epithelial cells. In numerous studies, Src has been described to be activated only during early phases of H. pylori infections, and delayed c-Abl activation maintained CagA phosphorylation at later time points (13–15, 17). In MEC1 cells, induction of a 60-kDa SFK member was observed after 2 h of infection with H. pylori, which remained stable throughout the infection, while a constitutively phosphorylated 50-kDa SFK member was immediately dephosphorylated in H. pylori-infected MEC1 cells. The identities of the individual SFK members are unknown; therefore, it is not clear which members of the SFK family, besides c-Src, are regulated in H. pylori-infected B cells and which of these kinases target CagA directly. In contrast to the situation in epithelial cells, we observed rapid and substantial activation of c-Abl in MEC1 cells, and this suggests that SFK and Abl kinases are simultaneously active. Since c-Src and c-Abl also target different EPIYA motifs in the CagA molecule in gastric epithelial cells (17), the consequences of these differentially regulated kinase activities on distinct EPIYA motifs have to be addressed in future studies.

However, it became evident that both tyrosine kinases are important for CagA phosphorylation as we could demonstrate that inhibition of individual SFK or Abl kinases only partly reduced CagA phosphorylation, while the inhibition of both kinase families by dasatinib was necessary to block the phosphorylation of CagA completely. Similar results were obtained in U937 cells, indicating that SFKs and c-Abl are important in monocytic host cells as well. In the context of MALT lymphoma, especially c-Abl activity appears to be of high interest since Craig and colleagues proposed that the progression from Helicobacter-associated gastritis to low-grade MALT lymphoma is accompanied by epigenetic silencing of the microRNA miR-203 that leads to an upregulation of c-Abl (39). In MEC1 cells, we also observed an increase of c-Abl upon H. pylori infection. In combination with an induced kinase activity, c-Abl phosphorylated CagA and actively deregulated associated signal transduction pathways. In accordance with the fact that treatment of freshly isolated CLL cell samples with dasatinib targeting SFK and Abl kinase activities is correlated with apoptosis induction (48), we conclude that pharmacological inhibition of SFKs and Abl kinases might represent an attractive candidate target for an alternative intervention in late-stage gastric MALT lymphoma or after failure of H. pylori eradication.

## Supplementary Material

Supplemental material
