# Supplementary material for: CagA Phosphorylation in Helicobacter pylori-Infected B Cells Is Mediated by the Nonreceptor Tyrosine Kinases of the Src and Abl Families
Source: Infect Immun. 2016 Aug 19;84(9):2671–80. doi: 10.1128/IAI.00349-16 (PMC4995908; doi:10.1128/IAI.00349-16)
Supplement: Supplemental material [file supp_84_9_2671__index.html]

Supplemental material 

# CagA Phosphorylation in Helicobacter pylori-Infected B Cells Is Mediated by the Nonreceptor Tyrosine Kinases of the Src and Abl Families

## Supplemental material

- Supplemental file 1 -

  Fig. S1. Specificity of the *in vitro* kinase assay monitoring c-Src activity. Fig. S2. Specificity of the *in vitro* kinase assay monitoring c-Abl activity. Fig. S3. CagA phosphorylation in MEC1 cells treated with 0.1 μM dasatinib.

  PDF, 2.0M
- Supplemental file 2 -

  Legends for Fig. S1 to S3. Table S1. Mammalian cell lines.

  PDF, 353K
