# Supplementary figures and images for: CagA Phosphorylation in Helicobacter pylori-Infected B Cells Is Mediated by the Nonreceptor Tyrosine Kinases of the Src and Abl Families
Source: Infect Immun. 2016 Aug 19;84(9):2671–80. doi: 10.1128/IAI.00349-16 (PMC4995908; doi:10.1128/IAI.00349-16)

Figure S1

A

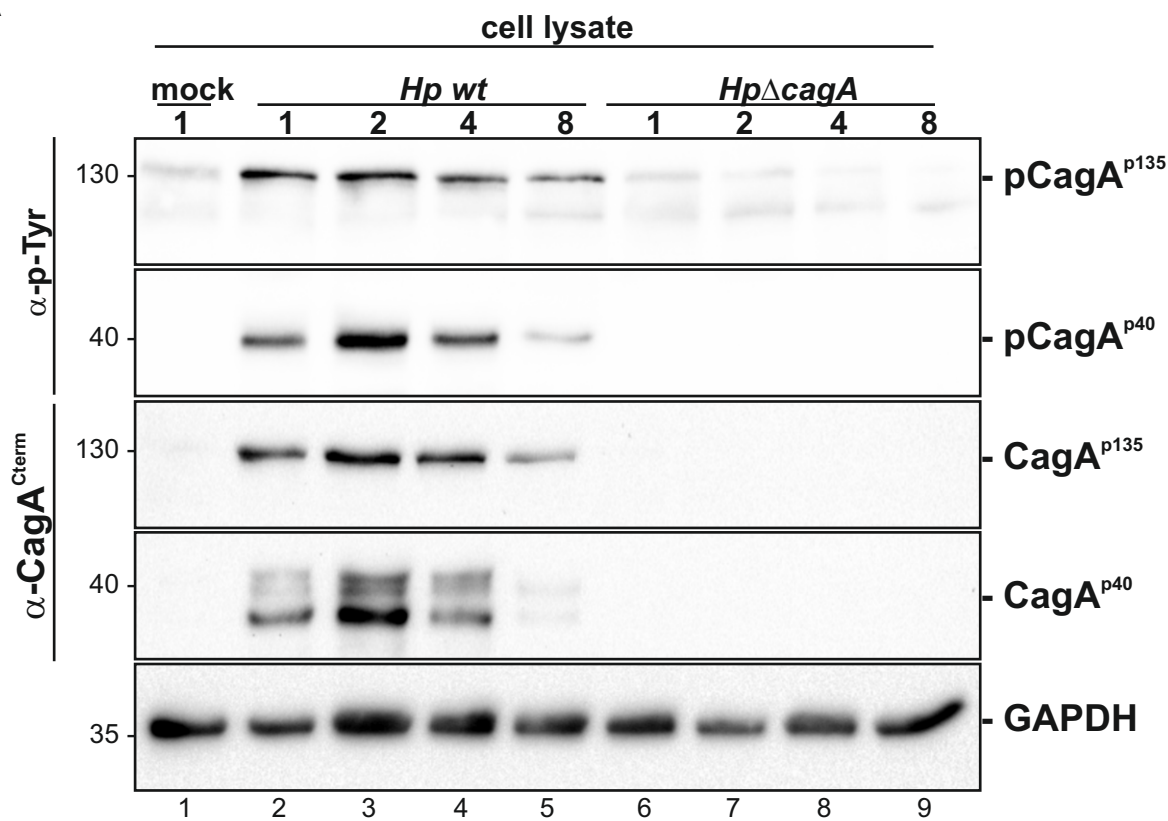

B

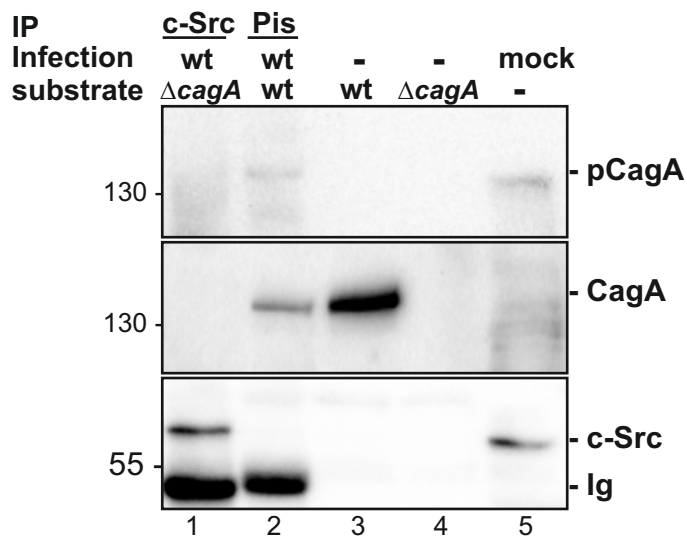

**A**

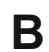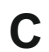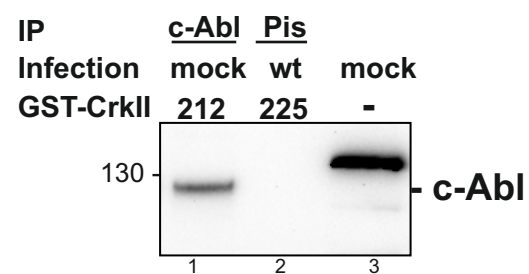

Figure S3

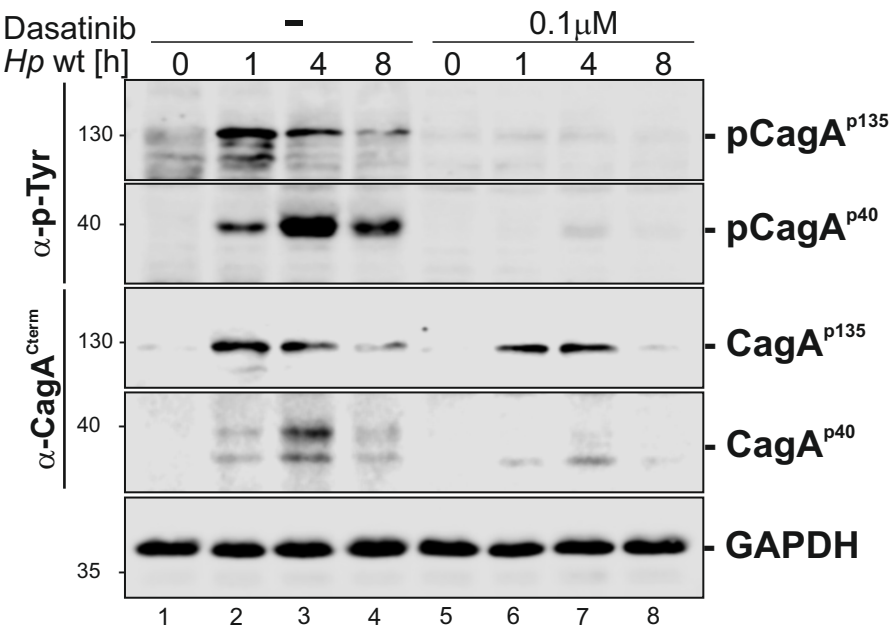

Supplement: Supplemental material [file IAI.00349-16_zii999091813so1.pdf]
