# Supplementary material for: CagA Phosphorylation in Helicobacter pylori-Infected B Cells Is Mediated by the Nonreceptor Tyrosine Kinases of the Src and Abl Families
Source: Infect Immun. 2016 Aug 19;84(9):2671–80. doi: 10.1128/IAI.00349-16 (PMC4995908; doi:10.1128/IAI.00349-16)
Supplement: Supplemental material [file IAI.00349-16_zii999091813so2.pdf]

Supplementary Information

CagA phosphorylation in *Helicobacter pylori*-infected B cells is mediated by the non-receptor tyrosine kinases of the Src and Abl family

Linda M. Krisch[1], Gernot Posselt[1], Peter Hammerl[2], Silja Wessler[1*]

[1]Cancer Cluster Salzburg, Department of Molecular Biology, Division of Microbiology, Paris-Lodron University, Salzburg, Austria. [2]Department of Molecular Biology, Division of Allergy and Immunology, Paris-Lodron University, Salzburg, Austria.

Figure legends

Fig. S1. Specificity of the *in vitro* kinase assay monitoring c-Src activity. (A) Cell lysates of the experiment shown in Fig. 3B were tested for phosphorylated CagA (pCagA^{p135} and pCagA^{p40}) using an anti-phospho-tyrosine antibody (α-p-Tyr) and total CagA (CagA^{p135} and CagA^{p40}) using an antibody directed against the C-terminus of CagA (α-CagA^{Cterm}). GAPDH is shown as a loading control. **(B)** Immunoprecipitation (IP) was performed using lysates of *H. pylori*-infected (wt) MEC1 cells using a polyclonal c-Src antibody (c-Src) or rabbit pre-immune-serum (Pis). Immunocomplexes were incubated with 10 µg *Hp* wt (wt) or *Hp*Δ*cagA* (Δ*cagA*) lysate in kinase buffer as substrates as indicated. Phosphorylated CagA (pCagA), total CagA and c-Src were analyzed by immunoblotting. Presented sections are from the same Western blot membranes as shown in Fig. 3B.

Fig. S2. Specificity of the *in vitro* kinase assay monitoring c-Abl activity. (A) Cell lysates of the experiment shown in Fig. 3C were analyzed for phosphorylated CagA (pCagA^{p135} and pCagA^{p40}) and total CagA (CagA^{p135} and CagA^{p40}). Asterisks indicate unspecific detection of a tyrosine

phosphorylated protein. GAPDH is shown as a loading control. **(B)** Immunoprecipitation was performed using cell lysates of uninfected (mock) or *H. pylori*-infected (wt) MEC1 cells using a monoclonal c-Abl antibody (c-Abl) or mouse pre-immune-serum (Pis). Immunocomplexes were incubated with 1 µg GST-CrkII aa120-225 (225) or GST-CrkII aa120-212 (212) that lacks Tyr^{221}, which is targeted by c-Abl. Phosphorylated GST-CrkII (pCrkII) and GST-CrkII were analyzed by immunoblotting. Presented sections are from the same Western blot membranes as shown in Fig. 3C. **(C)** Aliquots of immunoprecipitated c-Abl prior to the *in vitro* phosphorylation reaction were tested for efficient precipitation.

Fig. S3. CagA phosphorylation in MEC1 cells treated with 0.1 µM dasatinib. MEC1 cells were treated with 0.1 µM dasatinib prior to infection with *H. pylori* or remained untreated (-). Whole cell lysates were analyzed by immunoblotting using an anti-phospho-tyrosine antibody (α-p-Tyr) to detect phosphorylated full length CagA (p-$CagA^{p135}$) and the C-terminal CagA fragment (p-$CagA^{p40}$). A monoclonal anti-CagA antibody recognizing the C-terminal part of CagA (α-$CagA^{Cterm}$) was applied to show full length and fragmented CagA ($CagA^{p135}$, $CagA^{p40}$). As a loading control, the blot was reprobed with anti-GAPDH.

Table 1. Mammalian cell lines.

Cell line	Source[1] (Catalogue no.)	Cell type	Growth properties	Origin
**AGS**	ECACC (89090402)	epithelial	adherent	Gastric adenocarcinoma, caucasian female (54 yr)
**U937**	ATCC (CRL-1593.2)	monocyte	suspension	Histiocytic lymphoma, caucasian male (37 yr)
**MEC1**	DSMZ (ACC-497)	B cell	suspension	Chronic B cell leukemia, caucasian male (61 yr)

[1] ATCC, American Type Culture Collection (www.atcc.org); DSMZ, Deutsche Sammlung von Mikroorganismen und Zellkulturen GmbH (www.dsmz.de); ECACC, European Collection of Cell Cultures (www.ecacc.org.uk).
